# Decoupling of the PI3K Pathway via Mutation Necessitates Combinatorial Treatment in HER2+ Breast Cancer

**DOI:** 10.1371/journal.pone.0133219

**Published:** 2015-07-16

**Authors:** James E. Korkola, Eric A. Collisson, Laura Heiser, Chris Oates, Nora Bayani, Sleiman Itani, Amanda Esch, Wallace Thompson, Obi L. Griffith, Nicholas J. Wang, Wen-Lin Kuo, Brian Cooper, Jessica Billig, Safiyyah Ziyad, Jenny L. Hung, Lakshmi Jakkula, Heidi Feiler, Yiling Lu, Gordon B. Mills, Paul T. Spellman, Claire Tomlin, Sach Mukherjee, Joe W. Gray

**Affiliations:** 1 Oregon Health Sciences University, Department of Biomedical Engineering, Portland, Oregon, United States of America; 2 Lawrence Berkeley National Laboratories, Life Sciences Division, Berkeley, California, United States of America; 3 University of California San Francisco, Division of Heme/Onc, San Francisco, California, United States of America; 4 Netherlands Cancer Institute, Division of Biochemistry, Amsterdam, The Netherlands; 5 University of Warwick, Centre for Complexity Science, Coventry, United Kingdom; 6 University of California, Berkeley, Department of Electrical Engineering and Computer Sciences, Berkeley, California, United States of America; 7 MD Anderson Cancer Center, Department of Systems Biology, Houston, Texas, United States of America; The University of Texas MD Anderson Cancer Center, UNITED STATES

## Abstract

We report here on experimental and theoretical efforts to determine how best to combine drugs that inhibit HER2 and AKT in HER2^+^ breast cancers. We accomplished this by measuring cellular and molecular responses to lapatinib and the AKT inhibitors (AKT_i_) GSK690693 and GSK2141795 in a panel of 22 HER2^+^ breast cancer cell lines carrying wild type or mutant PIK3CA. We observed that combinations of lapatinib plus AKT_i_ were synergistic in HER2^+^/PIK3CA^mut^ cell lines but not in HER2^+^/PIK3CA^wt ^cell lines. We measured changes in phospho-protein levels in 15 cell lines after treatment with lapatinib, AKT_i_ or lapatinib + AKT_i_ to shed light on the underlying signaling dynamics. This revealed that p-S6RP levels were less well attenuated by lapatinib in HER2^+^/PIK3CA^mut ^cells compared to HER2^+^/PIK3CA^wt^ cells and that lapatinib + AKT_i_ reduced p-S6RP levels to those achieved in HER2^+^/PIK3CA^wt^ cells with lapatinib alone. We also found that that compensatory up-regulation of p-HER3 and p-HER2 is blunted in PIK3CA^mut^ cells following lapatinib + AKT_i _treatment. Responses of HER2^+^ SKBR3 cells transfected with lentiviruses carrying control or PIK3CA^mut^ sequences were similar to those observed in HER2^+^/PIK3CA^mut^ cell lines but not in HER2^+^/PIK3CA^wt ^cell lines. We used a nonlinear ordinary differential equation model to support the idea that PIK3CA mutations act as downstream activators of AKT that blunt lapatinib inhibition of downstream AKT signaling and that the effects of PIK3CA mutations can be countered by combining lapatinib with an AKT_i_. This combination does not confer substantial benefit beyond lapatinib in HER2^+^/PIK3CA^wt^ cells.

## Introduction

Genome aberration targeted therapies promise greater efficacy and fewer potential side effects than traditional chemotherapeutics for the treatment of advanced cancers. The efficacy of single targeted therapeutics has been less than hoped, especially in solid tumors [1, 2] but combination therapies have shown striking synergistic effects in subsets of patients [3–5]. This suggests the need to identify patient subsets that will benefit most from specific drug combinations. With the vast array of drugs available at present, it is not feasible to perform clinical testing of all possible combinations. Thus initial evidence for effective combination strategies typically comes from model systems such as cell line panels [6].

Approximately 20 percent of breast tumors are driven by amplification of HER2 (*HER2/ERBB2/NEU*) [7]. HER2-targeted drugs including the antibody based therapeutic trastuzumab and the dual EGFR/HER2 tyrosine kinase inhibitor lapatinib have improved outcomes for these patients in both the metastatic [8, 9] and adjuvant [10] settings, particularly when used in combination with chemotherapeutic agents such as capecitabine or letrozole [9, 11, 12]. More recently, combinations of lapatinib with trastuzumab have been shown to be synergistic in both experimental [4] and clinical settings [3], suggesting more complete inhibition of HER2 mediated signaling may be accomplished with combinations of pathway targeted drugs.

HER2 forms potent homodimers and heterodimers with HER3 to activate the PI3K-AKT and MAPK/ERK signaling pathways [13, 14]. Sergina *et al* showed that lapatinib induced inhibition of HER3 and PI3K-AKT signaling is transient [2]. As a consequence, inhibitors of PI3K-AKT signaling are being considered for the treatment of HER2^+^ tumors [15, 16]. Several reports suggest such combinations can be strongly synergistic [17–21] and clinical trials have been opened to study the efficacy of HER2 inhibitors in combination with AKT (NCT01245205) and PI3K inhibitors (NCT01471847 and NCT01589861).

Of course, not all HER2^+^ tumors respond equally well to HER2 and AKT targeted drugs. Several studies have shown that HER2^+^ tumors with mutations in *PIK3CA* or *PTEN* respond differently to HER2 targeted therapies than HER2^+^ tumor without these mutations [16, 18, 22]. Since *PIK3CA* mutations occur in about a third of HER2 amplified breast tumors [23], it is important to understand the impact of these mutations on response to combinations of drugs that target HER2 and AKT.

Based on this information, we tested the effect of PIK3CA mutations on cell and molecular responses to HER2 and AKT targeted inhibitors in 22 HER2^+^ human breast cancer cell lines. We used reverse-phase protein arrays (RPPA) to interrogate dynamic signaling responses in the cell lines. Our study showed that lapatinib in combination with AKT inhibitors was synergistic in HER2^+^ cell lines with *PIK3CA* mutations (HER2^+^/PKK3CA^mut^) but not in HER^+^ cells with wildtype PIK3CA (HER2^+^/PIK3CA^wt^). We validated this association by assessing responses of SKBR3 cells transfected with lentiviruses carrying control or mutant PIK3CA sequences. We developed a simplified dynamical model of AKT pathway signaling to show that our results are consistent with PIK3CA mutations stimulating AKT signaling downstream of HER2. In this model, lapatinib and AKT inhibitors are synergistic in HER2^+^/PIK3CA^mut^ cells because the combination is needed to suppress downstream AKT pathway activity that cannot be achieved using lapatinib alone.

## Materials and Methods

### Cell Lines

The cell lines AU565, BT474, HCC70, HCC202, HCC1419, HCC1569, HCC1954, MCF7, MDAMB175VII, MDAMB231, MDAMB361, MDAMB453, SKBR3, SUM190PT, SUM225CWN, UACC812, UACC893, and ZR75-30 have been previously described by us [24, 25] and are available from ATCC or DSMZ cell repositories or Asterand. We also added several new cell lines to increase the number of HER2^+^ cell lines available to us for study. EFM192A, EFM192B, EFM192C, and JIMT1 were all purchased from the DSMZ Biorepository, and were cultured in RPMI-1640 medium with 10% FBS (EFM lines) or DMEM with 10% FBS (JIMT1). 21MT1, 21MT2, 21NT, and 21PT have previously been described [26] and were kindly provided by K. Polyak from Dana Farber, and were cultured in DMEM supplemented with 10% FBS. All cell lines were genotyped and all matched genotypes available from Asterand, ATCC, and DSMZ. Five lines did not have published genotype information available; genotypes for these lines are listed in Supporting Information (SI) S1 Table.

### Drug Treatment

Drug treatments were carried out as previously described [25, 27], with several minor exceptions. Briefly, cell lines were treated in triplicate with each drug alone and in combination in a 1:1 molar ratio. There were nine two-fold dilutions tested for each drug/drug combination. Cells were plated into 96 well plates at densities that ensured that control cultures remained in the logarithmic growth phase during the whole assay. Cells were allowed to adhere overnight, and then were treated with drug. At the time of drug treatment, untreated cells plated at the same density were used to establish a time 0 reading (for calculations of GI_50_ and TGI) using the Cell Titer Glo (CTG, Promega) assay with a Biotek Luminometer. After 72 hours of incubation in the presence of drug, the samples were read using CTG and the luminometer to produce data on drug response. The drug treatment plates were set up in a “randomized” manner in order to minimize edge effects on the 96 well plates. Drugs used in the study included Lapatinib (Tykerb), and GSK690693 and GSK2141795 (AKT inhibitors), provided by GlaxoSmithKline. Additional Lapatinib and GSK690693 for the PIK3CA mutation studies were purchased from Selleck chemicals. The full drug response data that was used in conjunction with the RPPA data to generate models and identify significant proteins is available at the Synapse website (https://www.synapse.org/#!Synapse:syn2346643/wiki/232048).

### Drug Response Data Analysis

We fitted the drug response measurements with a sigmoidal curve were in order to estimate the concentration of drug needed to inhibit growth by 50% (GI_50_) as described [27]. These fits did not model the data well in cases where upper and lower plateaus were not present (because the drugs were tested in a narrower range than our single agent screens). We adapted the calculator for these cases to perform a piecewise linear fit through the data points. GI_50_ and TGI values were calculated based on the segment that crossed 50% and 100% growth inhibition according to NCI COMPARE methodology as previously described [25, 27]. The GI_50_ was calculated as 100 × (T−T0)/(C−T0) = 50 and TGI was calculated as 100 × (T−T0)/(C−T0) = 0 where T0 is the CTG luminescence at day 0, C is the untreated control CTG luminescence at day 3 and T is the CTG luminescence at the test concentration. There were several instances where the GI_50_ or TGI value was not crossed by a line segment, but instead lay between two line segments. In those instances, the GI_50_ or TGI value was assigned by taking the average of last points in each line segment. We calculated synergy using the median effect method of Chou and Talalay [28] to determine the combination index (CI) using IC_50_ values. We used an R code for the CI calculation that reports upper and lower 95% confidence intervals for the CI; we used the confidence intervals as a stringent cutoff to denote significant synergism or antagonism, such that significant synergism had an upper 95% CI less than 1 and significant antagonism had a lower 95% CI less than 1. For clustering and visualization of significant synergy, we transformed the CI as follows: if the CI was less than 0.8 and the upper 95% confidence interval was less than 1, it was assigned a value of -1, signifying significant synergy; if the CI was less than 0.8 but the upper 95% confidence interval was greater than 1, it was assigned a value of -0.25, (indicating a trend towards synergy); if the CI was between 0.8 and 1.2, it was assigned a value of 0; if the CI was greater than 1.2 but the lower 95% confidence interval was less than 1, it was assigned a value of 0.25; and if the CI was greater than 1.2 and the upper 95% confidence interval was greater than 1, it was assigned a value of 1, signifying significant antagonism. These values were then input into the cluster software algorithm [29] and subjected to median centering of the CI values for each drug dose followed by clustering of the cell lines using average linkage hierarchical clustering.

### RPPA Time Course and Data Analysis

Cells were plated into 10 cm^2^ dishes at a density of 1–2×10^6^ cells. After 24 hours, cells were treated with 250 nM lapatinib, 250 nM GSK690693, or a combination of the two, both at 250 nM. DMSO served as a control. Cells were harvested in RPPA lysis buffer at 30 min, 1h, 2h, 4h, 8h, 24h, 48h, and 72h post-treatment as described previously [30]. Cell lysates were quantitated, diluted, arrayed, and probed as described previously [30]. Imaging and quantitation of signal intensity was done as described previously [30]. For identification of significant protein levels that differed between synergistic (i.e., PI3K mutant) and non-synergistic (i.e., PI3K wild-type), statistical analysis was performed using R/Bioconductor [31] on the 11 cell lines known to be HER2^+^ by amplification (HCC70, MCF7, MDAMB231 and MDAMB175VII were excluded). For each of the 179 proteins, two mixed-model ANOVAs were performed with protein abundance as the dependent variable using the ezANOVA function ('ez' package). In the first, a combination of cell line and treatment were used as case identifier, time point entered as a within-subject factor, and synergy status and treatment entered as between-subject factors. In the second, cell line was used as case identifier, time point and treatment entered as within-subject factors, and synergy status as a between-subject factor. P-values were calculated for synergy, treatment, and treatment*synergy effects. P-values were corrected for multiple testing by Benjamini-Hochberg method with 'multtest' package. Significant genes were clustered using the Cluster/Treeview software [29] using average linkage clustering. A second time course was also generated on RPPA consisting of 174 validated proteins to analyze the specific effects of introduction of *PIK3CA* mutants into the wild type line SKBR3 (both full RPPA data sets that were used to generate the results are available as supplemental data).

### Dynamical model

An ordinary differential equation (ODE) model corresponding to a simplified HER signaling network was used to model AKT pathway dynamics and response to inhibition. The model was fitted to the RPPA time course data. We assumed Michaelis-Menten kinetics and allowed for the possibility of incomplete inhibition. Inference was carried out in a Bayesian statistical framework, using Markov chain Monte Carlo (MCMC) to obtain posterior probability distributions over model parameters. Predictions of AKT pathway activation were made using *maximum a posteriori* estimates. All computations were carried out using MATLAB R2009b. Further details concerning the dynamical model, including the system of ODEs, Bayesian formulation and MCMC, as well as additional empirical results, including checks of robustness to data perturbation and hyper-parameter specification, are provided in S1 methods.

### Retroviral Transduction

cDNAs encoding helical or kinase mutants of the *PIK3CA* gene were obtained from Frank McCormick (UCSF) in pENTR vectors and recombined with pBABE puro dest. Retroviral particles were prepared transient cotransfection of pBABE with pHIT60 and pVSV-G into 293T cells. Viral particles (supernatant) were collected at 48 hours post-transfection and used to infect SKBR3 cells growing in the log phase. Cells were transduced with mCherry containing vectors as a control. Cells were selected with 5 ug/ml puromycin until all non-transduced control cells were killed. Transduced cells were then maintained in 2 ug/ml puromycin until the time of drug treatment.

### Western Blotting

Western blotting was carried out as described previously [24]. Antibodies used included p-AKT S473, total AKT, p-4EBP1, total 4EBP1, p-S6RP, total S6, p-HER3, p-ERK, and p-PDK1 (all from Cell Signaling), and tubulin (Invitrogen) and actin (Santa Cruz) as controls. Images were captured using Bioluminescent imaging (Invitrogen) and x-ray film (Kodak) or with Alexa dyes and an Odyssey imaging system.

## Results

### Lapatinib synergizes with AKTi in HER2^+^/PIK3CA^mut^ cell lines

We measured responses to drugs targeting HER2 and AKT administered singly and in combination in 22 *HER2* ^+^ amplified breast cancer cell lines (see Table 1 for list of cell lines). Lapatinib was tested in combination with two pan-AKT inhibitors: GSK690693 [32] and GSK2141795 that bind to the ATP-binding pocket of AKT1, 2, and 3. The doses to inhibit growth by 50% (GI_50_) and to achieve total growth inhibition (TGI) for both AKT inhibitors alone and in combination with lapatinib are presented in Table 1 for GSK690693 and in S2 Table for GSK2141795. Interestingly, both AKT inhibitors showed responses that were correlated with response to lapatinib alone (see S1 Fig). This is consistent with the notion that effective inhibition of PI3K-AKT signaling by lapatinib in *HER2* ^+^ cells is one of the major requirements for therapeutic response.

**Table 1:** Average GI_50_ and TGI values for lapatinib in 22 HER2 positive breast cancer cell lines.

Cell Line	Ave GI_50_ (uM)	St Dev GI_50_	Ave TGI (uM)	St Dev TGI
21-MT1	>5	N/A	>5	N/A
21-MT2	>5	N/A	>5	N/A
21-NT	>5	N/A	>5	N/A
21-PT	>5	N/A	>5	N/A
AU565	0.171	0.075	0.448	0.170
BT474	0.090	0.058	0.364	0.508
EFM192A	0.253	0.149	0.948	1.000
EFM192B	0.898	0.417	>5	N/A
EFM192C	1.235	1.399	4.016	2.380
HCC1419	0.159	0.062	0.302	0.053
HCC1569	0.379	0.115	4.166	1.329
HCC1954	0.558	0.329	3.960	2.117
HCC202	0.125	0.123	0.293	0.236
JIMT1	>5	N/A	>5	N/A
MDAMB361	0.607	0.757	2.953	2.314
MDAMB453	0.807	0.567	4.801	0.526
SKBR3	0.144	0.112	0.599	0.424
SUM190PT	0.593	0.452	2.065	1.149
SUM225	0.209	0.156	1.775	2.249
UACC812	0.114	0.095	0.260	0.205
UACC893	0.254	0.190	0.661	0.633
ZR75-30	0.038	0.027	0.098	0.070

**Fig 1:**
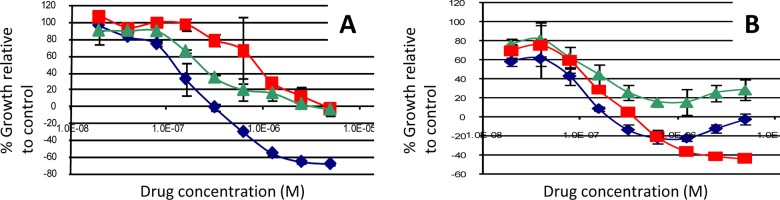
Examples of A. synergistic (in SUM190PT) and B. antagonistic (in AU565) interactions between lapatinib (red curve) and GSK690693 (green curve) in breast cell lines. Combination treatment is shown in the blue curve.

**Fig 2:**
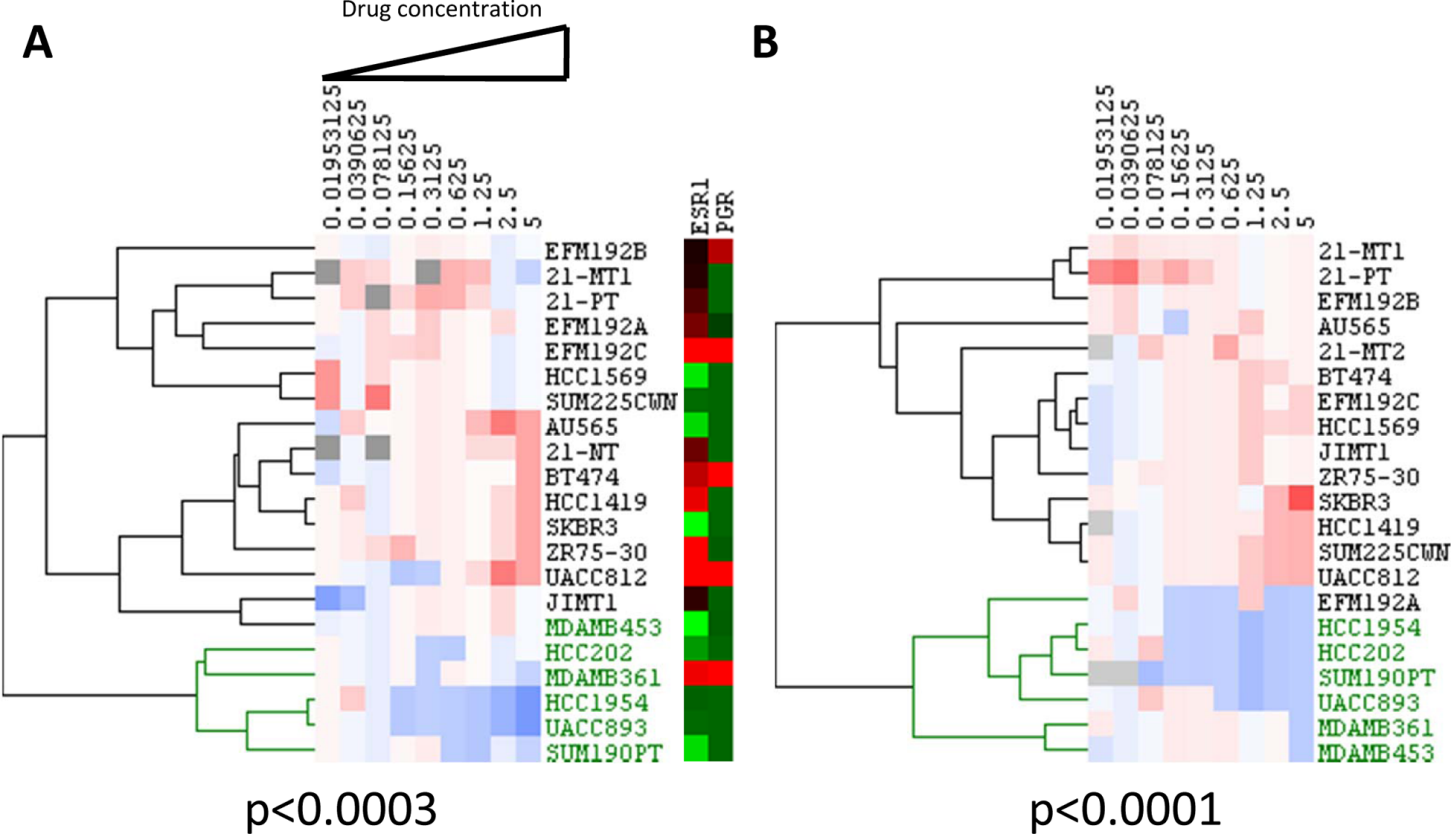
Combination index (CI) values were calculated for each of the nine dose combinations tested for each cell line and then clustered to generate synergy heatmaps for HER2 positive breast cancer cell lines treated with AKT inhibitors: A. GSK690693; B. GSK2141795. Blue indicates a significant synergistic CI value (CI<0.8, upper 95% confidence interval is less than 1), white indicates additivity, and red indicates significant antagonism (CI>1.2, lower 95% confidence interval is greater than 1). Gray represents instances where no CI could be calculated. A significant association between synergy and PI3K pathway mutation status was indicated by clustering, which was confirmed by Fisher’s exact test for **A** and **B**. Cell lines in green harbor hotspot PIK3CA mutations.

We initially examined a combination of lapatinib plus GSK690693 that was synergistic in some cell lines at multiple doses but additive or even antagonistic in others (see Fig 1A and 1B for representative examples; additional examples are available in S2 Fig). Responses in some cell lines were predominantly cytostatic—that is, there were more cells present than at the beginning of the assay, but fewer than in untreated controls. Drugs in other cell lines were cytotoxic, with fewer cells present at the end of the assay than at the start of the assay. We found no association between cytostatic responses versus cytotoxic response and synergism in the cell lines. We clustered the transformed CIs to identify cell lines in which the drug pairs were predominantly antagonistic and synergistic (see Materials and Methods; Fig 2A). Interestingly, 5/5 of the lines that had significant synergy also harbored hotspot mutations in *PIK3CA* (*P*<0.001 for the association between synergy and mutation status by Fisher’s exact test; see Fig 2A). Two of the synergistic lines harbored *PIK3CA* ^*E545K*^ (helical domain) mutations (MDAMB361 and HCC202) and three of the lines had *PIK3CA* ^*H1047R*^ (kinase domain) mutations (HCC1954, SUM190PT, and UACC893). In contrast, only 1/17 non-synergistic lines had a hotspot mutation in *PIK3CA*. The exception was MDAMB453 that had both an H1047R PIK3CA mutation and an inactivating *PTEN* mutation. This line showed responses to both lapatinib and GSK690693, but has one of the lowest levels of *HER2* amplification by copy number analysis [24], and some have questioned whether MDAMB453 truly qualifies as a HER2^+^ cell line.

Lapatinib plus GSK2141795 also produced a significant synergistic response in HER2^+^/PIK3CA^mut^ cells compared to HER2^+^/PIK3CA^wt^ cells (Fig 2B, *p*<0.001). Specifically, 6/6 HER2^+^/PIK3CA^mut^ cells showed a synergistic response while only 1 of 16 HER2^+^/PIK3CA^wt^ cells displayed synergy. The HER2^+^/PIK3CA^wt^ cell line, EFM192A, did show synergy with GSK2141795 and also displayed borderline significance with lapatinib plus GSK690693. The reason for the differential responses to the combinations using the two AKT inhibitors is unclear. One possibility is that EFM192A has a rare, non-hotspot C420R mutation in *PIK3CA* that may be activating and that may cause subtle differences in inhibitor binding or efficacy.

### Phospho-proteomics reveals signaling responses associated with synergy

We assessed changes in levels of signaling pathway proteins induced by treatment with lapatinib plus GSK69063 using Reverse Phase Protein Array (RPPA) analysis in order to identify pathways that played roles in synergistic and non-synergistic responses. We measured protein levels in samples collected at 30 min, 1h, 2h, 4h, 8h, 24h, 48h, and 72h after initiation of treatment for 15 cell lines (12 HER2 amplified, 1 HER2 over-expressing, and 2 non-HER2 amplified controls; see S3 Table). The HER2^+^ lines were chosen to be representative of both the synergistic (PIK3CA^mut^; N = 5) and non-synergistic (PIK3CA^wt^; N = 6) types. Cells were treated with 250 nM lapatinib, 250 nM GSK690693, or a combination of the two. These doses were within the range that showed synergistic interactions between the drugs and were close to the median GI_50_ values for responsive cells in the complete HER2^+^ cell line panel. S1 dataset lists the proteins assayed by RPPA and provides normalized protein levels for the time course.

We performed ANOVA analyses to identify differences in protein levels between synergistic (i.e., PIK3CA helical and kinase domain mutants) versus non-synergistic (i.e. PIK3CA^wt^) cell lines before and during treatment. Twenty-six proteins/phospho-proteins were significantly different between the two groups.

**Fig 3:**
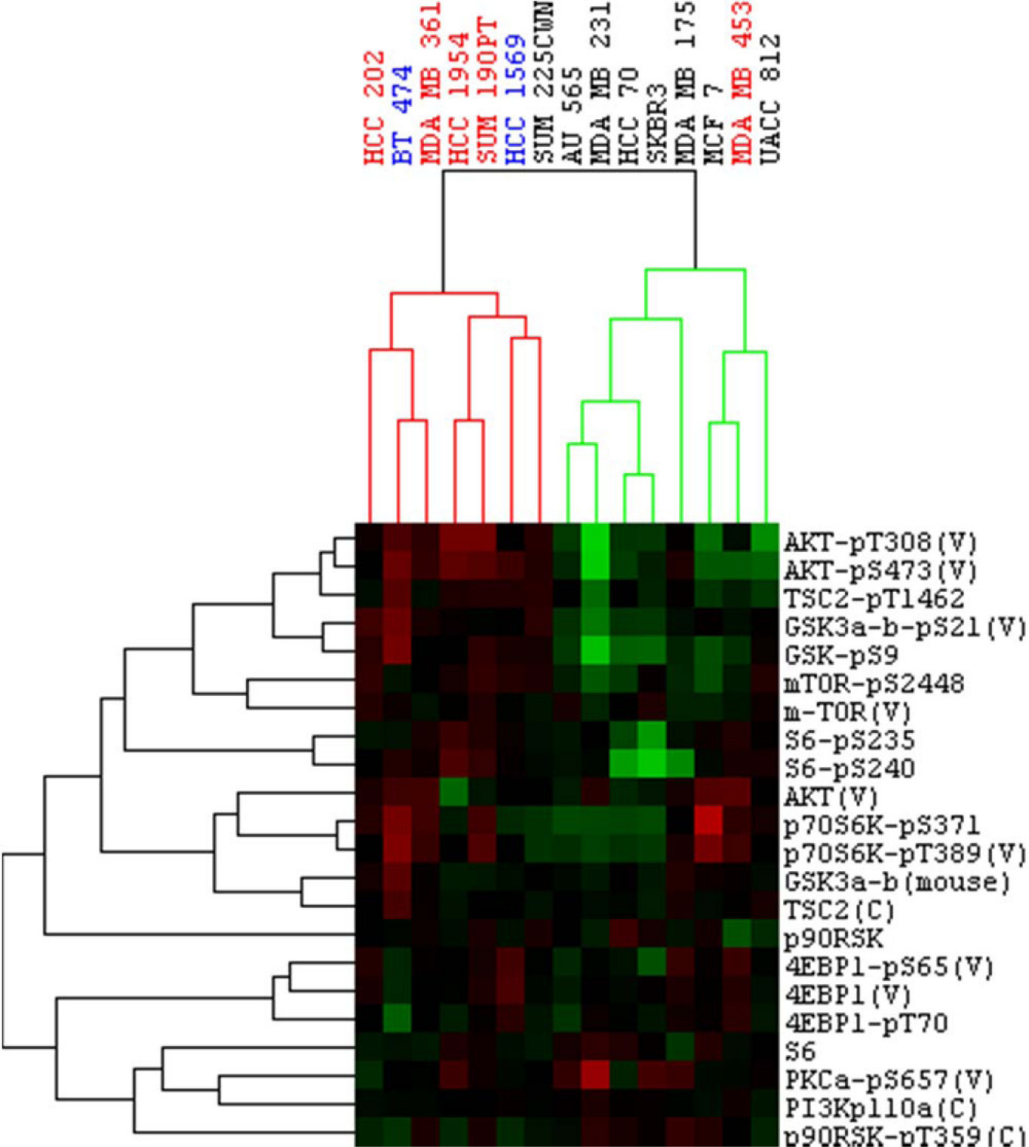
RPPA analysis of the PI3K-AKT pathway at baseline in PI3K mutant (red) and wild-type (black) HER2^+^ cell lines (*PTEN* mutant and K111N *PIK3CA* mutant lines shown in blue). MDAMB231, MCF7, and HCC70 are HER2^-^ cell lines. Heat map represents average expression for DMSO treated cells (baseline), with red being relatively high levels of expression, green being relatively low levels of expression. Different phospho-proteins shown include p-AKT (S473), p-AKT (T308), p-4EBP1 (S65), mTOR (S2448), p-p70S6K (S371), p-RPS6 (S235), all of which in general show higher levels of expression in the mutant PI3K, HER2 positive cell lines.

We observed several differences between protein levels in untreated cells. Notably, phosphorylation of AKT (at both Ser473 and Thr308) and other downstream proteins such as 4EBP1 (Ser65), mTOR (Ser2448), p70S6K (Ser371), and p-S6RP (Ser 235) were elevated in PIK3CA^mt^ cell lines. This is different from other studies that have reported that HER2^+^ breast tumors with PI3K mutations do not show elevated levels of PI3K pathway activity at baseline [23, 33]. Clustering of the cell lines based on baseline PI3K pathway protein levels separated them into two main clusters (Fig 3) that were composed of cells with mutations in the PI3K pathway and those without. The exception to this was MDAMB453, which did not cluster with the other HER2^+^/PIK3CA^mut^ lines. As noted above, this line did not show significant synergy with the combination of lapatinib plus GSK690693 and has borderline levels of HER2 amplification.

**Fig 4:**
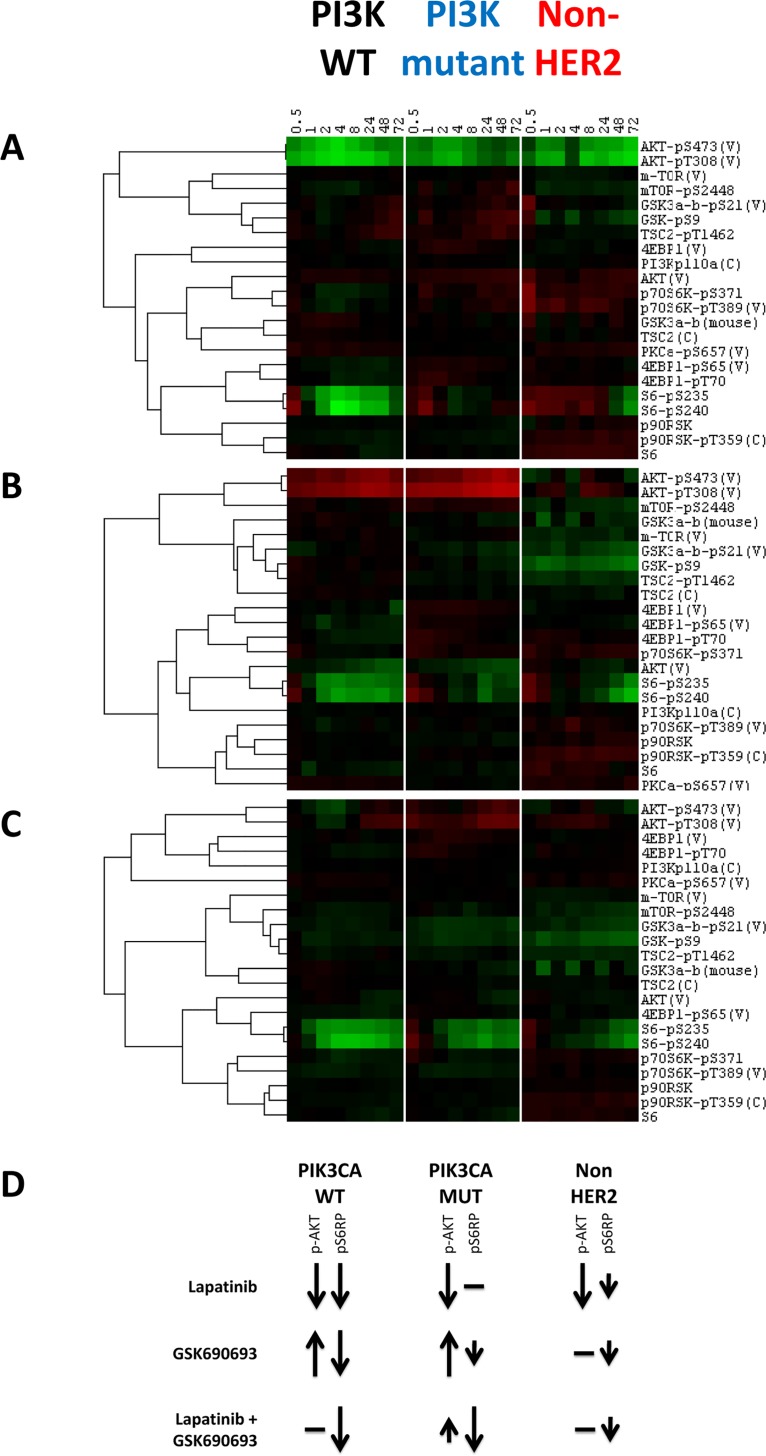
Heat maps showing average response within subtype (HER2^+^/PIK3CA^WT^; HER2^+^/PIK3CA^mut^; HER2^-^) to targeted therapeutics alone and in combination from RPPA data. **A**. Down-regulation of the PI3K-AKT pathway is evident in all HER2^+^ cells treated with lapatinib, irrespective of PI3K mutation status. However, the degree of down-regulation is less in cell lines with PI3K mutations (see p-S6 Ser235 for example). **B**. Treatment with GSK690693 leads to down-regulation of proteins downstream of AKT in all HER2^+^ cell lines, although the degree of down-regulation is less in lines harboring PI3K mutations. **C**. Treatment with the combination of lapatinib plus GSK690693 leads to down-regulation of the PI3K-AKT signaling pathway in both PIK3CA^mut^ and PIK3CA^WT^ lines. Note the much greater degree of down-regulation of S235 and S240 p-S6 in PIK3CA^mut^ cell lines treated with the combination relative to the single therapies. **D**. A summary of the most significant changes observed in the RPPA data for the PIK3CA^WT^, PIK3CA^mut^, and non-HER2 amplified cell lines under inhibition with lapatinib, GSK690693, or a combination of the two.

Several protein level changes occurred in all cell lines following treatment with lapatinib alone. These included down-regulation of phosphoproteins in the PI3K-AKT signaling pathway, including p-AKT (Ser473 and Thr308), p-S6RP (Ser235 and Ser 240), and p70S6K (Ser 371); see Fig 4A. These protein levels recovered over time as previously reported [2] though none appeared to recover to pre-treatment levels within 72 hours (Fig 4A). Other known PI3K-AKT pathway targets such as pGSK3 (Ser21 and Ser9) and TSC2 (Thr1462) also were significantly down-regulated, albeit of lesser magnitude. While the down-regulation of these phosphoproteins occurred in both synergistic and non-synergistic cell lines, we consistently observed that the magnitude of protein level changes was higher in the synergistic cell lines (Fig 4A). Other phosphoproteins involved in related signaling pathways that showed down-regulation and recovery, included SHC (Tyr317), MAPK (Thr202), STAT5 (Tyr694), and STAT6 (Tyr641) (data not shown).

Protein level changes in cell lines treated with GSK690693 alone were similar to those observed with lapatinib treatment, with several exceptions. Most notably, p-AKT (Ser473 and Thr308) increased in response to treatment (Fig 4B), a response that is consistent with treatment with kinase domain inhibitors of AKT [34], which block the activity of the kinase domain and also inhibit dephosphorylation of the protein. This leads to an increase in p-AKT levels but down-regulation of downstream members of the pathway. The increase in p-AKT pT308 levels was quite durable in both synergistic and non-synergistic lines. In contrast, p-AKT pS473 levels were more variable, but in general showed an initial increase followed by a slow decline. As expected, the phosphorylation of other proteins downstream of AKT in the pathway [e.g., p-RPS6 (Ser235 and Ser 240), p-70S6K (Ser 371), pGSK3 (Ser21 and Ser9), and p-TSC2 (Thr1462)] were decreased (Fig 4B), in a manner similar to the effects of inhibition with lapatinib. Interestingly, we also observed up-regulation of p-MAPK (Thr202) in both wild-type and mutant PI3K cell lines following treatment with GSK690693 (Fig 4B), consistent with previous results showing feedback regulatory loops between the MEK PI3K-AKT pathways [35].

The treatment of lines with the combination of lapatinib plus GSK690693 altered several protein levels in ways that differed from those produced by either inhibitor alone (Fig 4C and 4D). The p-AKT (Ser473 and Thr308) levels increased but were less than those observed when treating with the GSK690693 in isolation. This may represent interplay between the inhibition of phosphorylation resulting from lapatinib treatment and the loss of phosphatase activity as a result of GSK690693 treatment. Several of the cell lines showed virtually no change in p-AKT levels when treated with the combination, presumably due to these offsetting mechanisms of action. The most notable change was significant down-regulation of the levels of p-S6RP at both the Ser235 and Ser240 sites in both cell types. Unlike with lapatinib monotherapy, there was little recovery over time, and the difference between the synergistic and non-synergistic lines in absolute levels was minimal. Also, the combination prevented the feedback up-regulation of p-MAPK and p-MEK that was observed with GSK690693 alone, consistent with inhibition of MAPK signaling by lapatinib (S3 Fig). Other changes that occurred only in response to the combination treatment included induction of p-RB (Ser807) and down-regulation of CCNB1, both of which occurred in both synergistic and non-synergistic lines (data not shown).

We confirmed several of the changes that we observed by Western blotting. For example, treatment with lapatinib lead to down-regulation of p-AKT (S473) p-ERK, and p-HER3, while GSK690693 treatment leadto up-regulation of p-AKT (see S4 Fig), consistent with what was observed by RPPA.

### Expression of mutant PIK3CA is sufficient to induce synergy between lapatinib and GSK690693

**Fig 5:**
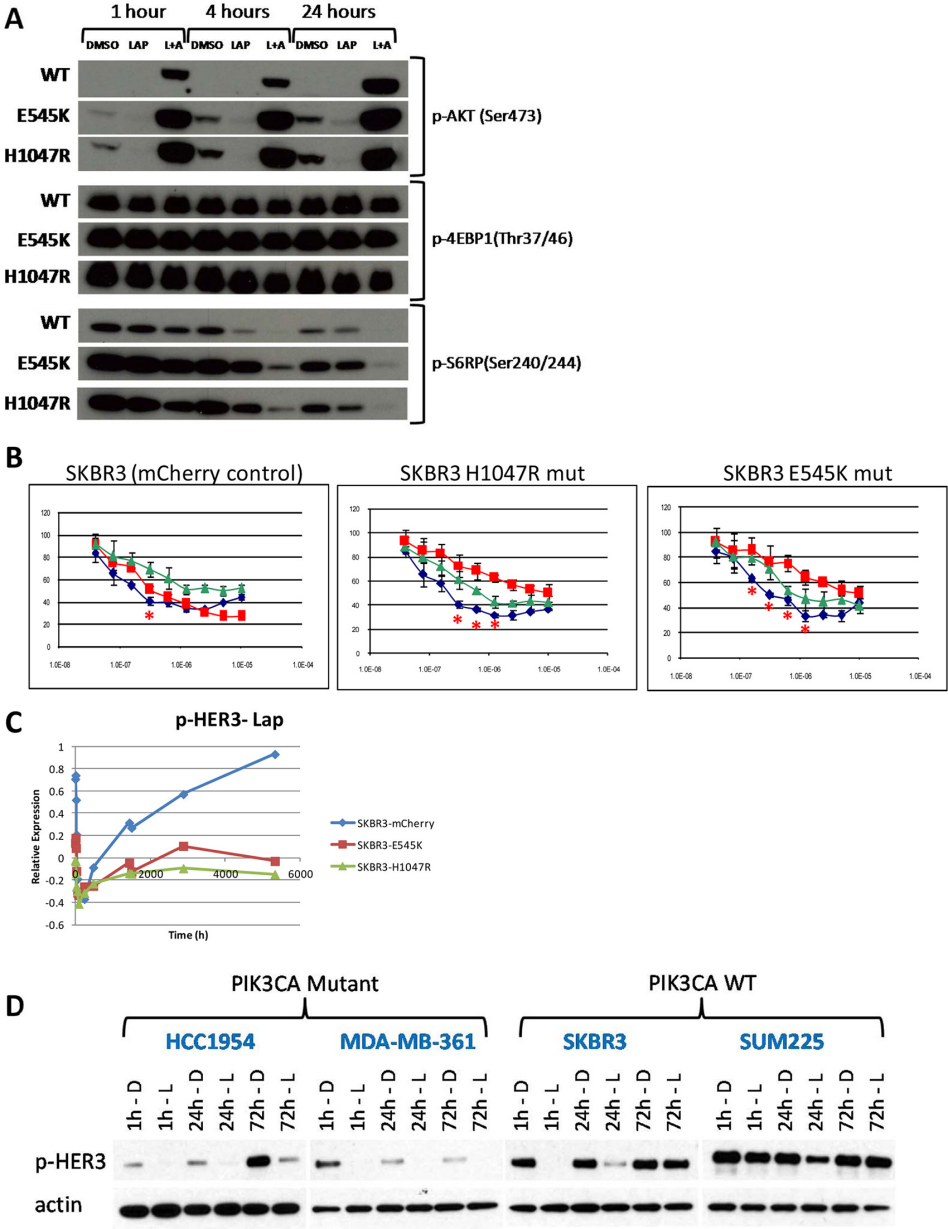
**A**. Western blots of SKBR3 cells transduced with retroviruses encoding either PIK3CA^*H1047R*^ or ^*E545K*^ mutant alleles or control vector (mCherry). Mutant transduced lines show increased levels of p-AKT and p-S6RP at baseline, and diminished response to lapatinib alone as measured by p-S6RP levels. Note that mutation has little effect on p-4EBP1 levels. **B**. Growth curves for control, E545K mutant, or H1047R mutant transduced SKBR3 cells treated with lapatinib (red), GSK690693 (green), or a combination of the two (blue). Introduction of mutations results in increased resistance to lapatinib and synergistic interactions between lapatinib and GSK690693 (doses with significant synergy are marked with a red asterisk), indicating a functional role for PIK3CA mutations in determining synergistic response to this combination. **C**. Analysis of RPPA time course data shows blunted recovery of p-HER3 levels in SKBR3 cells harboring PIK3CA mutations, while the control cell line shows the expected hyper-phosphorylation following lapatinib treatment. **D**. Western blotting confirms the lack of recovery in non-engineered cells lines with *PIK3CA* mutations compared to *PIK3CA* wild type lines.

We explored the hypothesis that *PIK3CA* mutations are causally linked to the synergistic response to lapatinib plus GSK690693 by introducing PIK3CA hotspot mutations (E545K or H1047R) into the *PIK3CA* wild type cell line SKBR3 by retroviral transduction. Immunoblotting showed that the introduction of the mutant PIK3CA increased baseline levels of p-AKT and p-S6RP relative to control (Fig 5A, DMSO treated cells). We treated the control and transfected cells with lapatinib or lapatinib plus GSK690693 as described above. The presence of either PIK3CA mutant decreased the ability of lapatinib to inhibit cell growth, diminished the lapatinib-induced down-regulation of p-AKT and p-S6RP and led to faster recovery to baseline levels. The presence of the mutations also rendered the lapatinib plus GSK690693 combination synergistic (Fig 5B). As described above, the synergistic interaction appeared to be due to a mutation-induced decrease in the response to lapatinib. Mutant harboring cells became more resistant to lapatinib and the addition of GSK690693 countered the mutation-induced resistance. As a consequence, response to the lapatinib-GSK690693 was approximately the same in PIK3CA wild type and mutant cells. Thus, the synergy resulted from the mutation-induced decrease in lapatinib sensitivity. We also performed an RPPA analysis of lapatinib and DMSO time course treatments, this time in SKBR3^mcherry^, SKBR3^E545K^, or SKBR3^H1047R^ cells (see S2 dataset for data). The RPPA measurements demonstrated that at baseline, there was little difference in the PI3K pathway activity between the cells (data not shown). However, with lapatinib treatment, there was a significantly greater down-regulation of PI3K pathway activity in the mCherry control cells compared to the mutant containing cells. Importantly, feedback up-regulation of p-HER3 (Fig 5C) and p-HER2 (not shown) in response to lapatinib was significantly blunted in the mutant cells. Western blot analysis of cell lysates from non-engineered *PIK3CA* ^*mut*^ cells confirmed that there was a reduced recovery of p-HER3 compared to *PIK3CA* ^*WT*^ cells following treatment with lapatinib (Fig 5D). This supports the idea that the mutation partially decouples the PI3K pathway from the surface receptors reducing the reliance on receptor mediated signaling for growth and blunting the response to receptor targeted therapies.

### Nonlinear dynamical modeling of mutant-specific synergy

**Fig 6:**
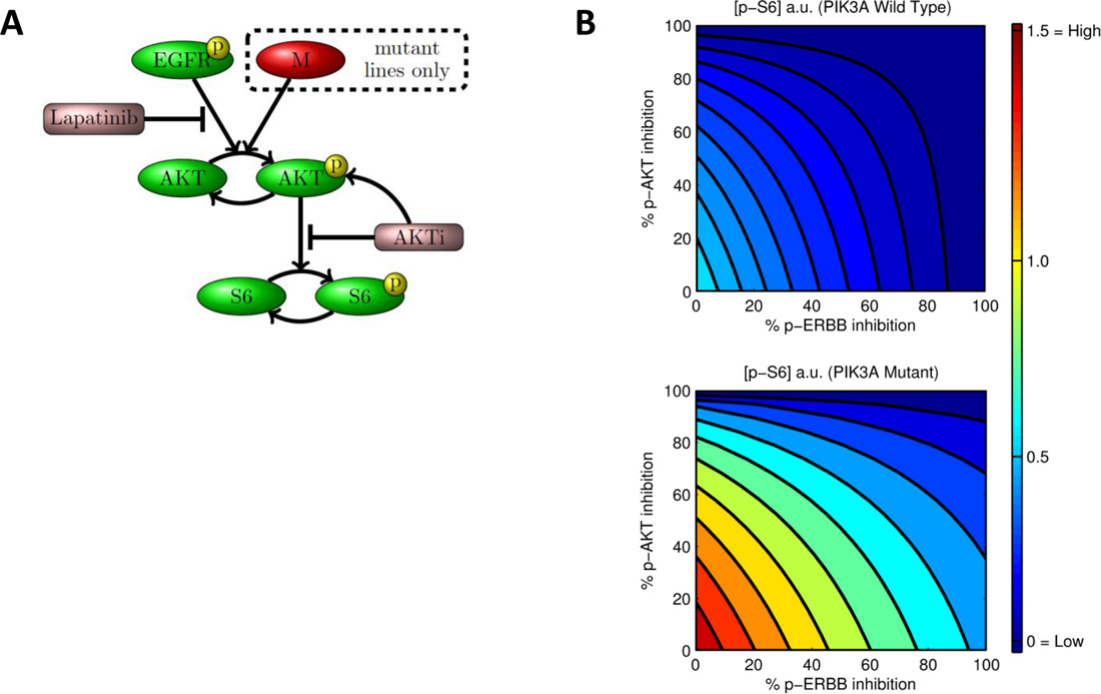
**A**. A simplified kinetic model for AKT pathway dynamics. Measured protein species (green; yellow circles indicate phosphorylation) are shown along with inhibitors (purple) and edges indicating regulatory interplay. An ERBB independent route to AKT activation is modeled as a node "M" (red) that can activate AKT in PIK3CA mutants only. To allow for the possibility that relevant player(s) were not observed in the RPPA data, the node was formally treated as an unobserved or latent variable. The edge from GSK690693 to p-AKT indicates that GSK690693 abrogates a negative feedback mechanism by which p-AKT regulates its own expression (possibly indirectly). All kinetic parameters were estimated using RPPA time-course data using a Bayesian statistical formulation. Note that links shown are not intended to represent direct influences but rather regulation via intermediate steps that are not explicitly modeled. **B**. AKT pathway activation (measured as equilibrium level of phospho-S6) as a function of the extent of ERBB (x-axis) and AKT inhibition (y-axis), as predicted by fitted model in **A**. In the wild type case (upper panel of **B**), ERBB inhibition alone is sufficient to abrogate S6 phosphorylation. This is evident by the rapid downregulation of p-S6 with increasing concentrations of lapatinib, such that by the time 80% of ERBB activity is inhibited, there is almost complete inhibition of p-S6. In contrast, PIK3CA mutant lines (lower panel of **B)**, which have on average higher p-S6 baseline activity than their wild type counterparts, show diminished effects on p-S6 activity with increasing concentrations of lapatinib monotherapy. Indeed, even when ERBB is completely inhibited, there is residual p-S6 activity present. In order to achieve full inhibition of p-S6, combined inhibition of ERBB and AKT is required. For example, to achieve full inhibition of p-S6, the model predicts that inhibition of both ERBB and AKT activity by at least 80% is required in the mutant lines.

We sought to understand whether PIK3CA mutations act simply as a downstream activator of HER2/3 signaling to produce the observed the observed changes in downstream pathway activity by fitting the response data using the nonlinear ordinary differential equation (ODEs) model in Fig 6A. We limited the model complexity to minimize over fitting. We modeled the effect of PIK3CA mutations as a simple bypass activation as illustrated. We estimated the model kinetic parameters from the previously described RPPA time-course (see S1 methods for details). Unfortunately, HER2 and HER3 levels were not measured in the initial RPPA assays, so we used EGFR (ERBB1) as a surrogate for HER2. We realize this is suboptimal but feel it is an acceptable strategy since EGFR is also inhibited by lapatinib and the protein phosphorylation kinetics of EGFR following lapatinib treatment mirrors those for HER2 and HER3 with an initial down regulation followed by recovery over time (see S2 dataset). Furthermore, we tested the sensitivity of the model to variation of the EGFR kinetic parameters and found that the model was relatively insensitive to variations in these parameters (see S1 methods).

We found that the model described the observed changes in pAKT and p-S6RP levels in both *PIK3CA* mutant and wild-type cell lines. Furthermore, the model allowed us to compute downstream AKT pathway activation (as quantified by predicted equilibrium level of p-S6RP) as a function of the extent of AKT and/or the ERBB tyrosine kinase receptor (EGFR/HER2) inhibition (Fig 6B). With lapatinib treatment alone, p-S6RP levels were reduced to near zero levels when the receptor was inhibited by 80% in wild type cells. In contrast, with lapatinib monotherapy, even 100% inhibition of the receptor in PIK3CA mutant cells did not result in complete abrogation of p-S6RP activity. Full inhibition of p-S6RP was only achieved when both AKT and the receptor were inhibited. These findings show that a subtle change to signaling network topology associated with *PIK3CA* mutation status is capable of explaining the synergistic effect of GSK690693 and lapatinib within an otherwise unified dynamical model. The model supports the idea that the synergism between lapatinib and GSK690693 results from the ability of GGSK690693 to inhibit downstream AKT activity in PIK3CA mutant cells that cannot be achieved using lapatinib alone.

## Discussion

Combination therapies promise to unlock the full clinical potential of targeted therapies. However, the vast number of possible combination therapies precludes direct experimental testing of all possible combinations and several non-trivial hurdles will have to be overcome if their potential is to be realized. One specific challenge is that of understanding how synergy is modulated by biochemical responses and dynamics that are specific to genetic background. A more comprehensive and quantitative understanding of such responses, including signaling, will be crucial in developing approaches that can exploit high-throughput biochemical assays and associated computational models to efficiently identify a small number of promising combinations to take forward to drug response assays and thence to *in vivo* and clinical studies.

Here we sought to better understand the relationship between signaling and synergy in the specific context of combination treatment of HER2^+^ breast cancer cell lines by Lapatinib and AKT_i_. We observed that the combination of lapatinib with AKT inhibitors was synergistic predominantly in cells that also had PI3K pathway mutations. Furthermore, by carrying out comprehensive phospho-protein profiling of the cell lines, through time, and following treatment by Lapatinib, AKT_i_ and their combination, we were able to shed new light on signaling changes associated with the synergy. Previous studies have shown that PI3K mutants do not have elevated PI3K pathway activity compared to wild-type cells [33]. Our proteomic data showed that there were consistently higher levels of p-AKT and other activated downstream PI3K-AKT pathway proteins in HER2^+^/PIK3CA^mut^ compared to HER2^+^/PIK3CA^WT^ cells. Interestingly, this was not maintained in the SKBR3 transduced cells, which did not show significant baseline differences by RPPA. However, significant differences were observed between mutant and wild type cells in response to lapatinib using both the cell line panel and the engineered SKBR3 cells. We found that introduction of a mutant form of *PIK3CA* resulted in decreased responsiveness to lapatinib but led to synergistic interactions between lapatinib and GSK690693, which corresponded to increased baseline levels of p-AKT and downstream PI3K pathway signaling in the mutant harboring cells. Intriguingly, our analysis of the data indicates that PIK3CA mutations decreased the efficacy of lapatinib, with the addition of GSK690693 reversing the resistance effect of the PIK3CA mutation and restoring the normal response to lapatinib.

With respect to lapatinib monotherapy, PI3K mutation status was not a major predictor of response. Several of the most resistant lines are wild type for PI3K, suggesting that alternative mechanisms are present that can dominate response above and beyond the relatively modest effects of PI3K mutations. Indeed, this is consistent with our results with the retrovirally transduced SKBR3 subclones, which demonstrated modest increases in GI_50_ with the introduction of PI3K mutations, but not frank resistance.

The RPPA data point to subtle alterations in the cells in response to the combination of lapatinib plus GSK690693. Down-regulation of elements of the PI3K-AKT pathway was notably less in cell lines with mutant *PIK3CA* compared to their wild-type counterparts when treated with lapatinib or alone. However, with the combination, there was much greater down-regulation of the pathway in mutant lines than was observed with the monotherapy, such that the response more closely mimicked that observed for PI3K wild-type lines. We speculate that in the absence of PI3K pathway mutations, lapatinib is sufficient to block the signal from the surface and shut down growth, through inhibition of both PI3K-AKT and MEK pathways. In HER2^+^/PIK3CA^mut^ cells, the presence of the mutation allows PI3K-AKT signaling to continue and thus results in decreased efficacy of the drug as manifested by elevated levels of PI3K signaling. When combined with GSK690693, both the signal from the surface and the signal propagating from the mutant PIK3CA are blunted, resulting in greater efficacy.

A dynamical model, incorporating a PIK3CA mutant-specific route to AKT activation, was able to explain the signaling dynamics as observed in RPPA experiments, in both mutant and wild-type cell lines. Furthermore, model-based predictions of AKT pathway activation levels as a function of extent of inhibition supported the notion of greater efficacy for combination inhibition in the PIK3CA mutant background. The simplified model we propose provides an example where a subtle, mutation-associated change to network wiring explains a substantive difference in response. In particular, the model demonstrates that a hypothesis of mutant-specific regulation of AKT activation is plausible in terms of observed signaling dynamics. However, it is important to note that while the model is sufficient to explain core pathway dynamics and response to dual inhibition it represents only a minimal, schematic description of signaling. In particular, the model cannot by itself elucidate the mechanism underlying the putative regulation but rather should be regarded as a coarse description that could be consistent with a number of specific mechanisms for synergy. The key causal hypothesis encoded in the model is that the *PIK3CA* mutation confers specific regulation that in turn leads to synergy. To experimentally test the putative causal link between *PIK3CA* mutation and synergy we asked whether introduction of *PIK3CA* mutations into wild-type lines could change response patterns from non-synergistic to synergistic. Indeed, we found that mutations did alter response and render the combination synergistic, verifying the causal hypothesis. However, further work will be needed to understand the precise biochemical events that lead from mutation to synergy.

It is important to note that several of the non-synergistic lines harbored other mutations in *PIK3CA* that have been previously observed at a much lower frequency. The first of these was BT474, which harbors a K111N mutation in *PIK3CA*. This mutation has been reported to confer weak oncogenicity, unlike the other *PIK3CA* mutations, which are strongly transforming in chick embryo fibroblasts [36]. Similarly, JIMT1 and the EFM192 lines all harbor another *PIK3CA* mutation (C420R). Interestingly, EFM192A showed significant synergy with GSK2141795. The C420R mutations has been shown to confer oncogenicity in colony forming assays, although it was less potent than the common helical and kinase domain hotspot mutations [36]. More importantly, these assays were performed in chick embryo fibroblasts, and similar oncogenicity studies have not been reproduced in mammalian cells, making it unclear what the functional significance of these alterations is in human cells.

Lapatinib has proven to be an effect treatment for patients with HER2^+^ breast cancer when used in combination with chemotherapeutic agents [9] or trastuzumab [3, 4], but not as a monotherapy. It is thought that much of the efficacy of lapatinib occurs through the inhibition of the PI3K pathway, a notion supported by recent studies that have suggested a potential role for combination therapies of lapatinib with PI3K targeted therapeutic agents [16, 17, 22]. Several of these studies have noted that mutations in PIK3CA or PTEN are associated with resistance to HER2 targeted therapeutics or poor outcomes in HER2^+^ patients [16, 18]. Our results point to an important distinction that may be specific to HER2^+^ cells. Several recent studies have suggested that combinations of receptor tyrosine kinase inhibitors (such as lapatinib) and PI3K pathway inhibitors are an effective means of treatment to counteract re-activation of PI3K pathway signaling elicited by activation of secondary or compensatory pathways [12, 17]. In contrast, our results suggest that there may be a threshold of PI3K pathway activity that must be overcome in HER2^+^ cells with *PIK3CA* mutations that can be suppressed with AKT inhibitors, resulting in synergistic interactions with lapatinib. The lack of response (and even evidence of antagonistic interaction) in cell lines with wild-type *PIK3CA* with such combinations speaks against a role for feedback related pathway activation and resistance to therapy in such cells, as has been proposed for a mechanism of response to TKI treatments alone [2, 12, 17]. Indeed, such pathway re-activation may be a general phenomenon, as we and others have observed up-regulation of MEK pathway proteins as well following an initial down-regulation after lapatinib treatment. The mechanism that causes the antagonism we observed in the wild-type cell lines between lapatinib and the AKT inhibitors is unclear, but is an interesting observation that we are currently following up on.

The recent publication of the TCGA analysis of breast cancer genomic landscape indicates that there seem to be two HER2^+^ subtypes, one of which appears to be luminal in nature while the other is more consistent with ER negative tumors [23]. *PIK3CA* mutations are reported to be prevalent in both groups, suggesting that combination therapies may be effective in subsets of both of the HER2^+^ groups observed in TCGA. Our analysis of the TCGA HER2^+^ subtypes indicates that many of the features of these two subsets are recapitulated in vitro in the cell lines.

In conclusion, we observed a significant synergistic interaction between lapatinib and AKT inhibitors in HER2 positive breast cancer cell lines when those lines also harbor PI3K pathway mutations. We present new proteomic time-course data that sheds light on the synergy, allowing us to build a simplified dynamical model that explains the mutant-specific synergy. Our data suggests that PIK3CA mutations decouple or buffer the pathway from the surface receptors, rendering them less responsive to inhibition of the receptor. The clinical implications of our work are twofold. First, our findings suggest that clinical trials treating breast cancer with lapatinib in combination with AKT (e.g., NCT01245205) or PI3K inhibitors (e.g., NCT01471847 and NCT01589861) should pay careful attention to PIK3CA status, as our data predicts patients with PIK3CA mutations are most likely to benefit from such combination treatment regimes. Second, our results and approach suggest that bringing together relevant biochemical assays and associated dynamical models may in the future enable rational prioritization of promising combination therapies that are specific to genetic background.

## Supporting Information

S1 Dataset Normalized, log2 transformed, and centered RPPA time course data for the first RPPA study of a panel of 15 breast cancer cell lines. (XLSX)

S2 Dataset Normalized, log2 transformed, and centered RPPA time course data for the SKBR3 mutant cell lines. (XLSX)

S1 Fig Correlation between Lapatinib and AKTi response. Correlation between GI50 values calculated for: **A** GSK690693 and AKTi GSK2141795 and **B** lapatinib and AKTi.
(TIF)

S2 Fig Representative dose response curves for treated cells. Cells were treated with lapatinib (red), GSK690693 (green), or a combination of the two drugs (blue). Cells in the left column showed a synergistic interaction of the combination, while there was limited benefit to the cells in the right column.
(TIF)

S3 Fig Inhibition of MAPK feedback activation with combinatorial treatment. Feedback activation of MEK-MAPK signaling following treatment with GSK690693 (upper panel) is blunted when the combination of lapatinib plus GSK690693 is used (lower panel).
(TIF)

S4 Fig Western blot validation of RPPA data. Analysis of key PI3K-AKT signaling pathway molecules in the cell line SKBR3 confirm the changes in protein levels observed in the RPPA data.
(TIF)

S1 Methods Supporting Information for dynamical modeling methodology. (PDF)

S1 Table Genotyping information for cell lines lacking genotyping information in public repositories. (XLSX)

S2 Table GI50 and TGI values (in uM) for lapatinib, GSK690693, GSK2141795, Lapatinib plus GSK690693, and Lapatinib plus GSK2141795 in the HER2+ cell line panel. (XLSX)

S3 Table Cell lines used in RPPA study with HER2, PIK3CA, and PTEN status. (XLSX)
