# Correction: Decoupling of the PI3K Pathway via Mutation Necessitates Combinatorial Treatment in HER2+ Breast Cancer

**DOI:** 10.1371/journal.pone.0186551

**Published:** 2017-10-11

**Authors:** James E. Korkola, Eric A. Collisson, Laura M. Heiser, Chris Oates, Nora Bayani, Sleiman Itani, Amanda Esch, Wallace Thompson, Obi L. Griffith, Nicholas J. Wang, Wen-Lin Kuo, Brian Cooper, Jessica Billig, Safiyyah Ziyad, Jenny L. Hung, Lakshmi Jakkula, Heidi Feiler, Yiling Lu, Gordon B. Mills, Paul T. Spellman, Claire Tomlin, Sach Mukherjee, Joe W. Gray

The middle initial of the third author is missing. The third author’s correct name is: Laura M. Heiser. The correct citation is: Korkola JE, Collisson EA, Heiser LM, Oates C, Bayani N, Itani S, et al. (2015) Decoupling of the PI3K Pathway via Mutation Necessitates Combinatorial Treatment in HER2+ Breast Cancer. PLoS ONE 10(7): e0133219. https://doi.org/10.1371/journal.pone.0133219
