# Supplementary figures and images for: Decoupling of the PI3K Pathway via Mutation Necessitates Combinatorial Treatment in HER2+ Breast Cancer
Source: PLoS One. 2015 Jul 16;10(7):e0133219. doi: 10.1371/journal.pone.0133219 (PMC4504492; doi:10.1371/journal.pone.0133219)

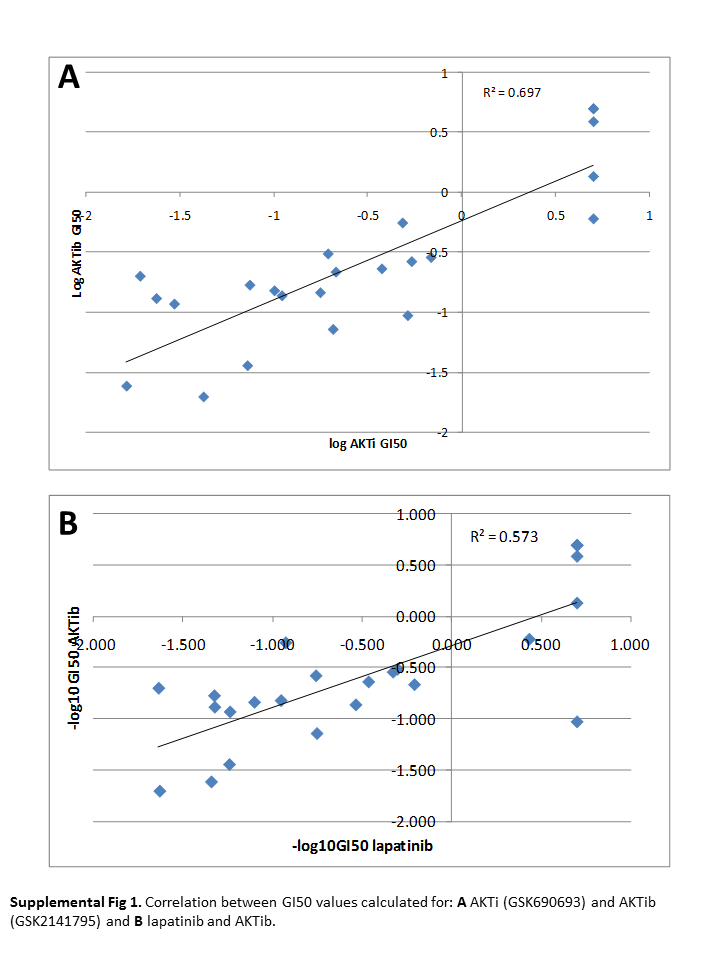

Supplement: S1 Fig — Correlation between GI50 values calculated for: A GSK690693 and AKTi GSK2141795 and B lapatinib and AKTi. (TIF) [file pone.0133219.s003.tif]

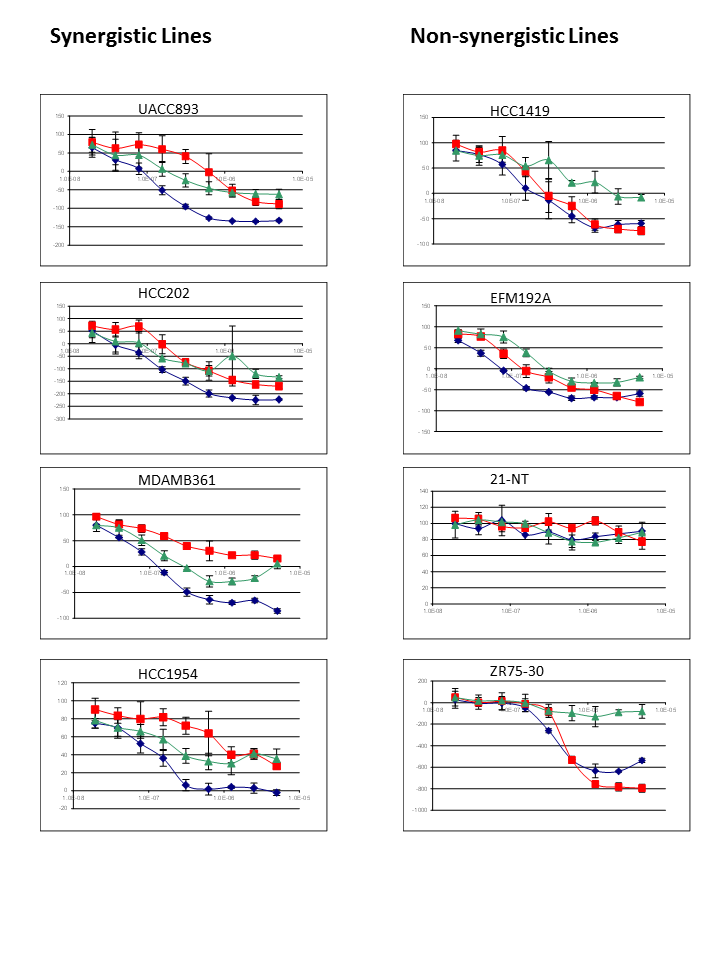

Supplement: S2 Fig — Cells were treated with lapatinib (red), GSK690693 (green), or a combination of the two drugs (blue). Cells in the left column showed a synergistic interaction of the combination, while there was limited benefit to the cells in the right column. (TIF) [file pone.0133219.s004.tif]

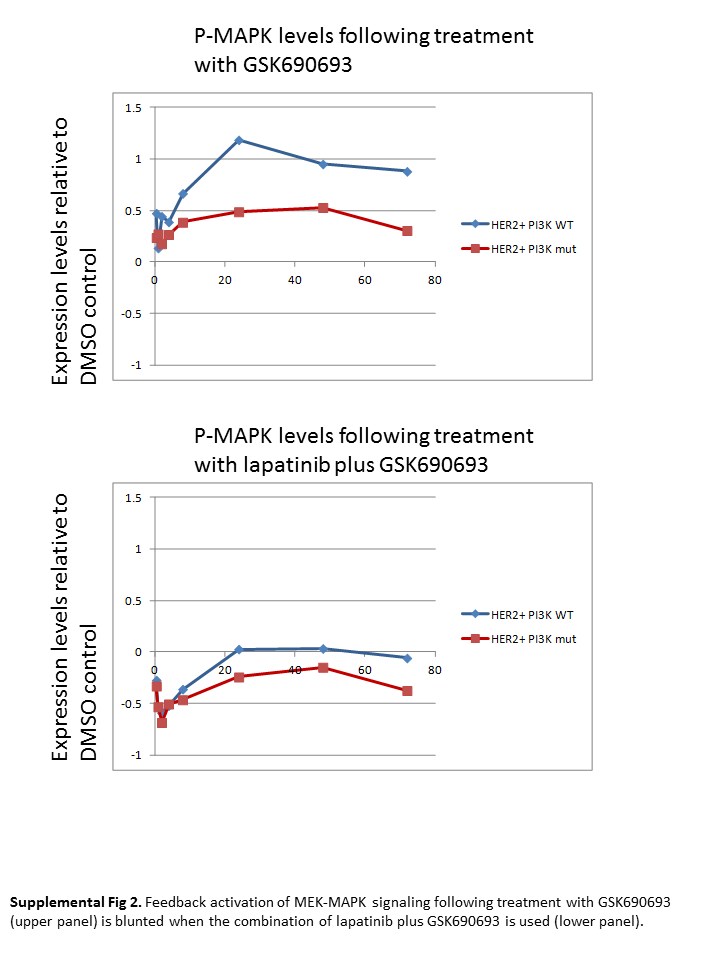

Supplement: S3 Fig — Feedback activation of MEK-MAPK signaling following treatment with GSK690693 (upper panel) is blunted when the combination of lapatinib plus GSK690693 is used (lower panel). (TIF) [file pone.0133219.s005.tif]

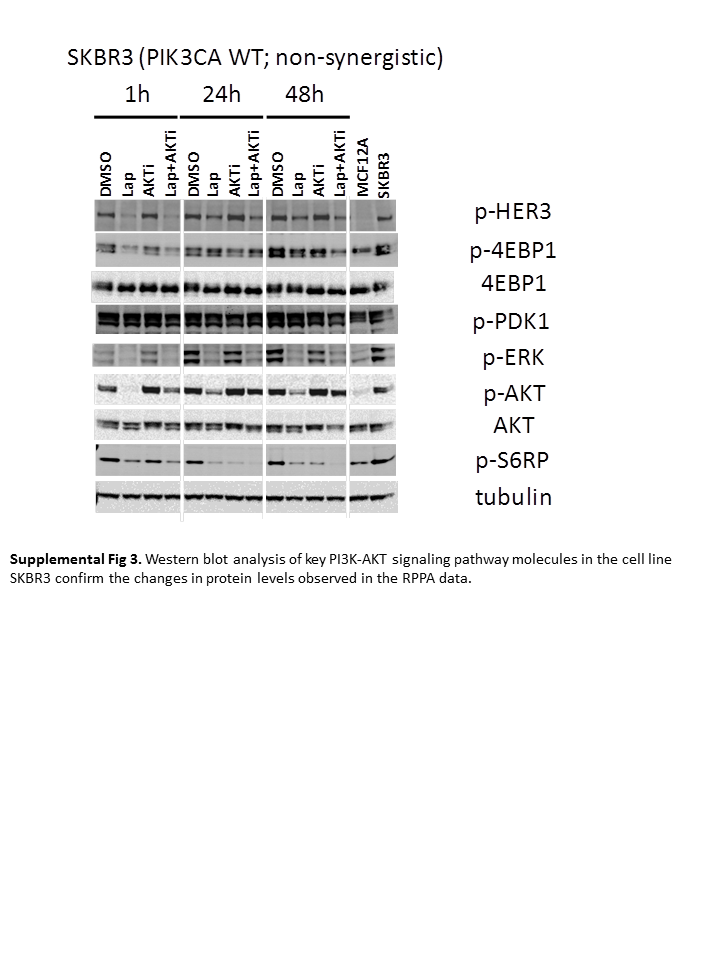

Supplement: S4 Fig — Analysis of key PI3K-AKT signaling pathway molecules in the cell line SKBR3 confirm the changes in protein levels observed in the RPPA data. (TIF) [file pone.0133219.s006.tif]
