# Supplementary material for: Decoupling of the PI3K Pathway via Mutation Necessitates Combinatorial Treatment in HER2+ Breast Cancer
Source: PLoS One. 2015 Jul 16;10(7):e0133219. doi: 10.1371/journal.pone.0133219 (PMC4504492; doi:10.1371/journal.pone.0133219)
Supplement: S1 Methods — (PDF) [file pone.0133219.s007.pdf]

Korkola *et al.*, "Decoupling of the PI3K Pathway via Mutation Necessitates Combinatorial Treatment in HER2+ Breast Cancer", Supplementary Information for dynamical modeling

Here we describe the dynamical model used to explore AKT pathway activation in PIK3CA mutant and wild-type cell lines, as outlined in Main Text and shown in Fig 5 therein.

Contents

1 Model **1**

2 Statistical inference **3**
2.1 Data and noise model 3
2.2 Bayesian formulation 4

3 Results **4**
3.1 Model fit and posterior distributions 4
3.2 Prediction of inhibition effects 4
3.3 Sensitivity analysis 5

4 References **5**

1 Model

We consider the signaling species shown in Fig 5A in Main Text, namely ERBB, AKT and S6. We assume that PIK3CA mutants differ from the wild-type case in having an ERBB independent route to AKT phosphorylation; otherwise we assume a unified dynamical model, both with respect to network topology and kinetic parameters. We do not make any assumption about the identity of the species mediating the mutant-specific AKT activation; to allow for the possibility that the relevant species are not included in the RPPA assay, in the statistical formulation below we treat the corresponding variable as a latent

variable. Specifically, we assume a species "M" activates AKT independently of ERBB, as shown in Fig 5A in Main Text. Note also that although the link is shown as between M and AKT, we do not assume that phosphorylation is direct, but rather that it may occur through intermediate steps that are not explicitly described in the model. In the same way, the links from ERBB to AKT and from AKT to S6 are not intended to describe direct influences. All model parameters are estimated from RPPA time-course data, using a Bayesian formulation described below.

Our model is presented graphically in Fig 5A in Main Text. In particular we do not model regulation of ERBB, which rapidly phosphorylates upon stimulation. We also assume that the fractions of phosphorylated protein are small relative to total protein, so that total protein levels may be used as a proxy for unphosphorylated protein levels. Then, conditional on the total protein levels $\boldsymbol{X}$ remaining constant, we describe the dynamics of phosphorylated species $\boldsymbol{X}^*$ using Michaelis-Menten kinetics (Kholodenko 2006, Leskovac 2003, Steijaert 2010). We allow for phosphatase-mediated dephosphorylation, but do not model the dynamics of phosphatases themselves.

Inhibitors (here, AKTi and Lapatinib) are modeled as reducing kinase activity of their targets. For simplicity we assume both inhibitors have negligible off-target effects. However inhibition is not assumed to be perfect (in the sense of completely removing kinase activity of the target); rather, a proportion $0 \leq \alpha \leq 1$ of the kinase activity is allowed to remain after intervention. Inhibitor data are modeled using indicator functions, as $[\text{p-AKT}] \mapsto \alpha^{\mathbb{I}(\text{AKTi})}[\text{p-AKT}]$ for example, where $\mathbb{I}(\text{AKTi}) = 1$ if the AKT inhibitor is used, otherwise $\mathbb{I}(\text{AKTi}) = 0$.

It has been observed that p-AKT levels increase under treatment with AKTi; indeed Engelman (2009) writes "AKT catalytic site inhibitors might not block AKT phosphorylation, and might increase its phosphorylation through loss of negative-feedback regulation of PI3K". We therefore introduce a simple linear feedback term $\gamma[\text{p-AKT}]$ into the model for p-AKT dynamics. This term is abrogated for experiments in which AKTi has been used, since then the kinase ability of AKT is blocked by the inhibitor, thereby removing the feedback. Note that we do not directly model the full mechanism underlying the feedback; rather the feedback term is intended to capture the overall dynamical influence on AKT phosphorylation. This term is indicated as a feedback edge in the network model (Fig 5A in Main Text).

Collecting together our modelling assumptions produces a kinetic description

of phosphorylation dynamics:

$$\frac{d[\text{p-ERBB}]}{dt} = 0. \tag{1}$$

$$\begin{aligned}\frac{d[\text{p-AKT}]}{dt} &= -\left(V_1\frac{[\text{p-AKT}]}{[\text{p-AKT}]+K_1} + \gamma\alpha_2^{\mathbb{I}(\text{AKTi})}[\text{p-AKT}]\right) \\ &\quad +V_2\alpha_1^{\mathbb{I}(\text{ERBBi})}[\text{p-ERBB}]\frac{[\text{AKT}]}{[\text{AKT}]+K_2} \\ &\quad +V_3\mathbb{I}(\text{mutant})\frac{[\text{AKT}]}{[\text{AKT}]+K_3}\end{aligned} \tag{2}$$

$$\frac{d[\text{p-S6}]}{dt} = -V_4\frac{[\text{p-S6}]}{[\text{p-S6}]+K_4} + V_5\alpha_2^{\mathbb{I}(\text{AKTi})}[\text{p-AKT}]\frac{[\text{S6}]}{[\text{S6}]+K_5}. \tag{3}$$

2 Statistical inference

We use a Bayesian approach to carry out inference for the model described above, using RPPA time course data (as described in the Main Text) to fit the model. Bayesian inference requires prior probability distributions $p(\boldsymbol{\theta})$ for model parameters $\boldsymbol{\theta}$. This distribution is updated in light of observed data to produce a posterior distribution $p(\boldsymbol{\theta}|\text{data})$ from which conclusions may be drawn. Below we present our prior specification and computational approach for obtaining the posterior distribution.

2.1 Data and noise model

Write $\boldsymbol{X} = ([\text{ERBB}], [\text{AKT}], [\text{S6}])$ for total protein concentrations and $\boldsymbol{X}^* = ([\text{p-ERBB}], [\text{p-AKT}], [\text{p-S6}])$ for phosphoprotein concentrations. We consider noisy measurements $\boldsymbol{Y}, \boldsymbol{Y}^*$ of protein concentrations $\boldsymbol{X}, \boldsymbol{X}^*$ obtained at discrete times $t_1, \ldots, t_m$ seconds. Further, we consider several experiments $E \in \mathcal{E}$, so that $\boldsymbol{X}_E^i(t_j)$ are the concentrations of protein i in experiment E at time t_j (similarly for $\boldsymbol{X}_E^{*i}$, $\boldsymbol{Y}_E^i$ and $\boldsymbol{Y}_E^{*i}$). Different experiments are characterized by different cell lines and different drug treatments. Total proteins levels $\boldsymbol{X}_E(t)$ at time t were approximated from data $\boldsymbol{Y}_E$ using linear interpolation, so that $\boldsymbol{X}_E(t) \approx \boldsymbol{Y}_E(t_j)\left(1 - \frac{t-t_j}{t_{j+1}-t_j}\right) + \boldsymbol{Y}_E(t_{j+1})\left(\frac{t-t_j}{t_{j+1}-t_j}\right)$, where $t_j < t \leq t_{j+1}$. We denote by $\boldsymbol{X}^*(t, \boldsymbol{x}^*)$ the solution, conditional upon $\boldsymbol{X}_E$ as above, to Eqns. 1,2,3 at time t, subject to the initial ($t = 0$) phosphoprotein concentrations $\boldsymbol{X}^* = \boldsymbol{x}^*$.

RPPA measurements are known only up to proportionality; we therefore normalized each species to have unit mean expression, where the average is taken across all experiments $\mathcal{E}$. RPPA measurement error induces an approximately log-normal distribution for the observations, for example $\log(\boldsymbol{Y}_E^i) \sim \mathcal{N}(\log(\boldsymbol{X}_E^i), \sigma^2\boldsymbol{I})$ where typical signal-to-noise ratio is $\sigma^{-1} \approx 10$. (Here and henceforth we define $\log(\boldsymbol{v})$ to be the vector with ith component $\log(v_i)$.)

2.2 Bayesian formulation

Write $\boldsymbol{\theta} = (\boldsymbol{V}, \boldsymbol{K}, \boldsymbol{\alpha}, \gamma)$ for all unknown parameters. Following Xu *et al.* (2010) we assume all processes occur on observable time scales, motivating weakly informative gamma priors $V_i \sim \Gamma(a,b)$, $K_i \sim \Gamma(c,d)$ where $a = 2$, $b = 1/2000$, $c = 2$, $d = 1/2$ were chosen to give prior means $\overline{V_i} = 1/1000$ a.u./sec, $\overline{K_i} = 1$ a.u.. (Here the shape, scale parametrization is used.) For the interventional effect we take a beta distribution $\alpha_i \sim \beta(e,f)$, where $e = 2$, $f = 5$ are chosen to give a prior drug efficacy of $\overline{\alpha_i} \approx 0.3$, meaning that 70% of kinase activity is abrogated as a result of the drug treatment. The effect γ is assigned $\gamma \sim \Gamma(a,b)$ since the effect on phosphorylation of AKT under treatment with AKTi is of a similar magnitude to that of p-ERBB inhibition. Initial phosphoprotein concentrations $\boldsymbol{x}^*$ are assumed *a priori* to be drawn from the empirical distribution of the protein-specific data, or rather, from a log-normal distribution $\log N(\boldsymbol{\mu}, \boldsymbol{\Sigma})$ with mean and covariance $\boldsymbol{\mu}$, $\boldsymbol{\Sigma}$, fit to this data.

Inference then proceeds based on the random variable $\boldsymbol{Y}^*|\boldsymbol{Y}, \boldsymbol{x}^*, \boldsymbol{\theta}$, using the relevant conditional densities collected together below:

$$
\begin{aligned}
p(\boldsymbol{x}^*) &= \prod_{E \in \mathcal{E}} \mathcal{N}(\log(\boldsymbol{x}^*_E); \boldsymbol{\mu}, \boldsymbol{\Sigma}) &(4)\\
p(\boldsymbol{\theta}) &= p(\boldsymbol{V})p(\boldsymbol{K})p(\boldsymbol{\alpha})p(\gamma) &(5)\\
&= \Gamma(\boldsymbol{V}; a, b)\Gamma(\boldsymbol{K}; c, d)\beta(\boldsymbol{\alpha}; e, f)\Gamma(\gamma; a, b) &\\
p(\boldsymbol{Y}^*|\boldsymbol{Y}, \boldsymbol{x}^*, \boldsymbol{\theta}) &= \prod_{E \in \mathcal{E}} \prod_{j=1}^{m} \mathcal{N}(\log(\boldsymbol{Y}^*_E(t_j)); \log(\boldsymbol{X}^*_E(t_j, \boldsymbol{x}^*_E; \boldsymbol{\theta})), \sigma^2 \boldsymbol{I}) &(6)
\end{aligned}
$$

The notation $\Gamma(\boldsymbol{V}; a, b)$ indicates that each component V_i of $\boldsymbol{V}$ is independently distributed as $\Gamma(a,b)$; we use similar notation for the beta distribution.

Markov chain Monte Carlo was used to sample from the posterior distribution. We exploited log-normal Metropolis-Hastings parameter proposals within a blocking strategy, using standard diagnostics to test convergence.

3 Results

3.1 Model fit and posterior distributions

Fig 1 displays parameter posterior distributions obtained under inference. Fig 2 demonstrates that the model is able to reproduce observed dynamics.

3.2 Prediction of inhibition effects

As shown in Fig 5B in Main Text, we predicted the effect of ERBB and/or AKT inhibition by considering the equilibrium level of S6 phosphorylation as a function of Lapatinib and AKTi efficiency. This was done using *maximum a posteriori* parameter values obtained after Bayesian inference as described above.

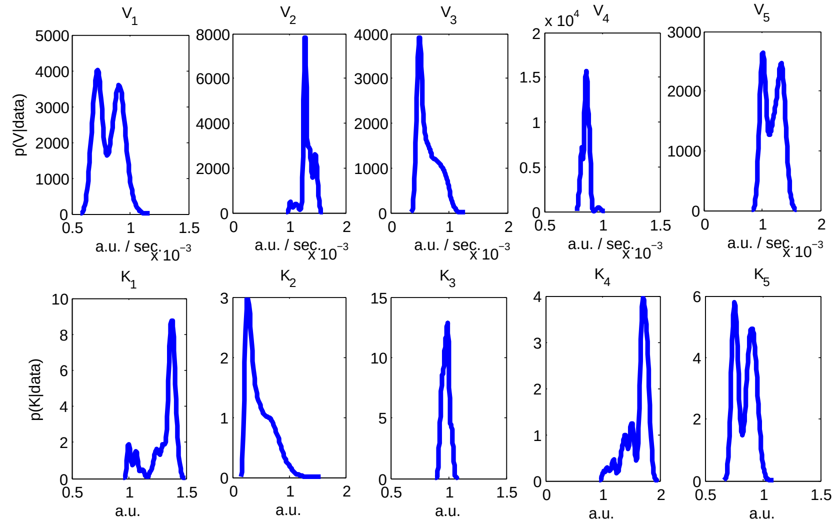

Figure 1: Parameter posterior distributions, obtained using Markov chain Monte Carlo. [Kernel density estimates (blue).]

3.3 Sensitivity analysis

In order to ensure our results were not exquisitely sensitive to the actual data or to the Bayesian statistical formulation, we repeated the prediction procedure of Section 3.2 under (i) perturbation of the actual data and (ii) perturbation of the hyperparameters a, b, c, d, e, f as defined in Section 2.2. For (i) we sequentially removed one time points from all experiments, performing prediction based only on the remaining 7 time points. Results in Fig 3 demonstrate that the quantitative results of Fig 5B in Main Text are extremely robust to deletion of single time points. For (ii) we considered each hyperparameter in turn, either halving or doubling values prior to prediction. Results in Fig 4 demonstrate that the qualitative result of Fig 5B in Main Text is robust to variation of hyperparameters, though the quantitative prediction of S6 phosphorylation does depend weakly upon the configuration of the Bayesian model. This latter finding is consistent with the parameter posterior distributions (Fig 1) in suggesting that further data are required for precise identification of model parameters.

4 References

1. Engelman, J.A. (2009). Targeting PI3K signalling in cancer: opportunities, challenges and limitations. *Nat Rev Cancer* **9**, 550-562.

2. Kholodenko, B. (2006). Cell Signalling Dynamics in Time and Space. *Nat Rev Mol Cell Bio* **7**(3), 165-176.

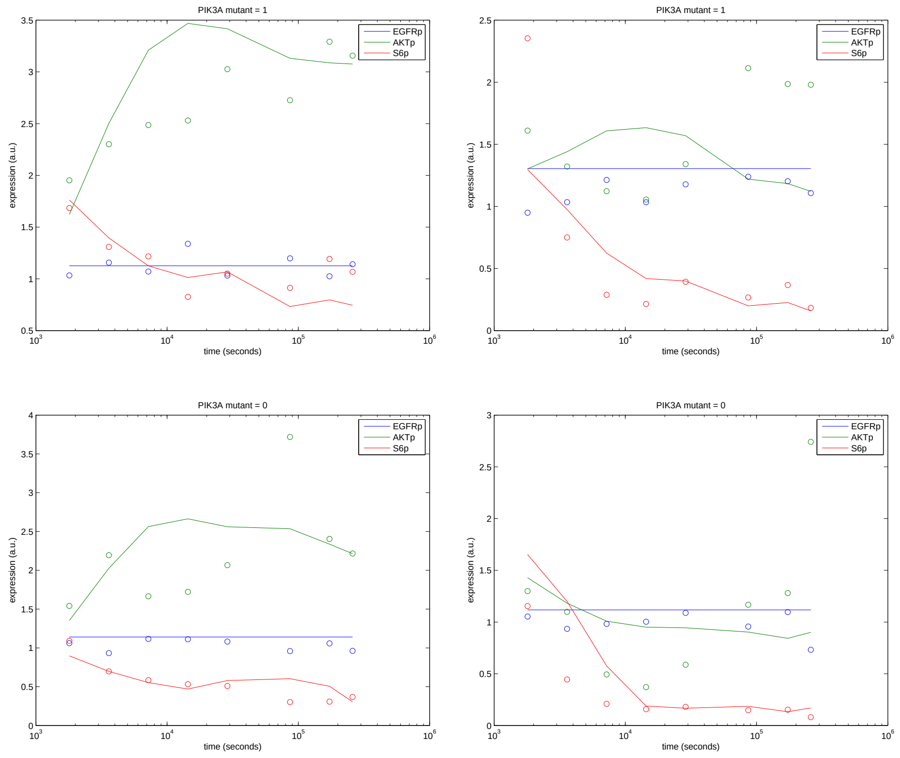

Figure 2: The proposed model is able to fit simultaneously data from both PIK3A wild-type and mutant cell lines. In total there are 44 time courses (11 lines $\times$ 4 drugs) for each protein, all of which must be fit by common parameters $\boldsymbol{\theta} = (\boldsymbol{V}, \boldsymbol{K}, \boldsymbol{\alpha}, \gamma)$. Here we display 4 such time courses (circles) with fitted trajectories (lines) corresponding to *maximum a posteriori* parameter values. [Top: PIK3CA mutant. Bottom: PIK3CA wild-type.]

3. Leskovac, V. (2003). Comprehensive Enzyme Kinetics. Kluwer Academic/Plenum Pub., New York.

4. Steijaert, M. *et al.* (2010). Computing the Stochastic Dynamics of Phosphorylation Networks. *J Comput Biol* **17**(2), 189-199.

5. Xu, T.R. *et al.* (2010). Inferring signaling pathway topologies from multiple perturbation measurements of specic biochemical species. *Sci Signal* **3**(113):ra20.

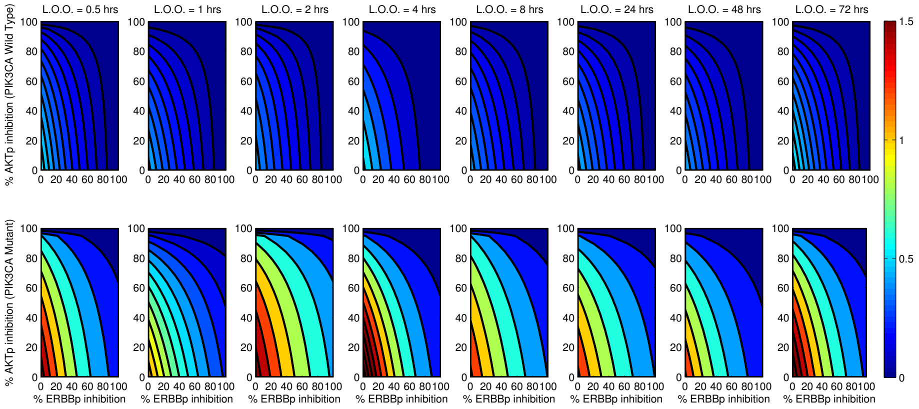

Figure 3: Sensitivity analysis; one time point was removed from all experiments and prediction was performed based only on the remaining 7 time points. [Colors are used to denote [p-S6] in arbitraty units. "L.O.O" = leave one out.]

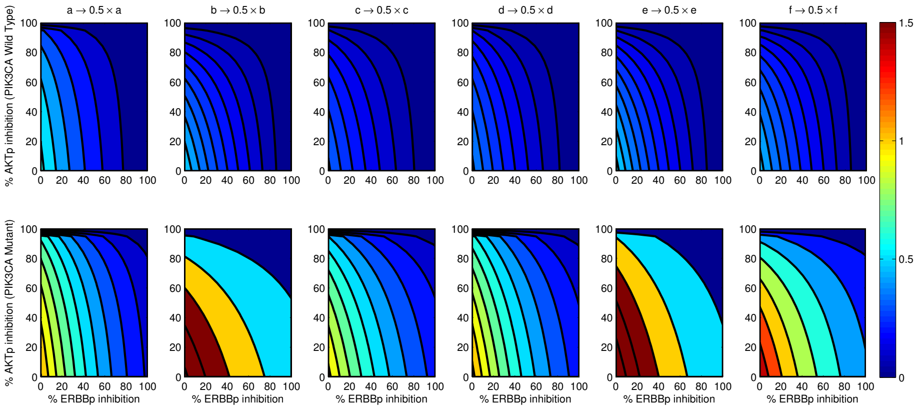

(a) Halving hypermarameter values

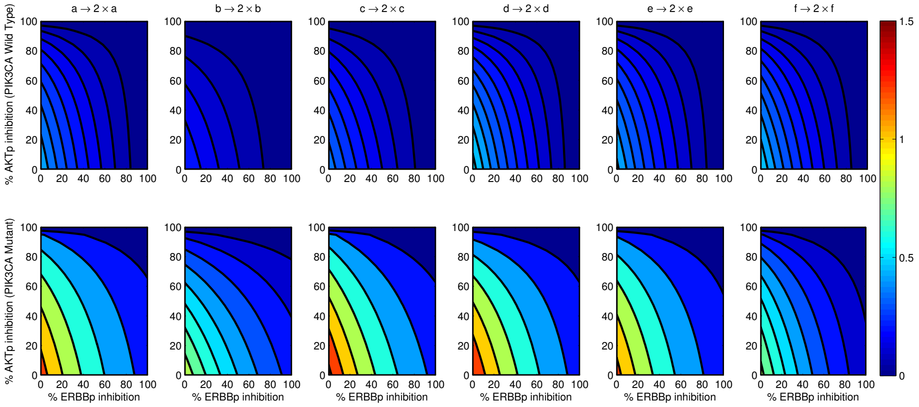

(b) Doubling hypermarameter values

Figure 4: Sensitivity analysis; the hypermarameters a, b, c, d, e, f were varied sequentially. [Colors are used to denote [p-S6] in arbitraty units. (a) hyperparameters were halved; (b) hyperparameters were doubled.]
